# Inhibition of Notch Signaling Attenuates Schistosomiasis Hepatic Fibrosis via Blocking Macrophage M2 Polarization

**DOI:** 10.1371/journal.pone.0166808

**Published:** 2016-11-22

**Authors:** Shaoping Zheng, Peige Zhang, Yixiong Chen, Shaojiang Zheng, Liping Zheng, Zhihong Weng

**Affiliations:** 1 Department of Ultrasound, Union Hospital, Tongji Medical College, Huazhong University of Science and Technology, Wuhan, China; 2 Division of Gastroenterology, Union Hospital, Tongji Medical College, Huazhong University of Science and Technology, Wuhan, China; 3 Department of Pathology and Key Laboratory of Molecular Tumor Pathology, Hainan Cancer Hospital, Affiliated Hospital of Hainan Medical College,Haikou, China; 4 Department of Infectious Diseases, Union Hospital, Tongji Medical College, Huazhong University of Science and Technology, Wuhan, China; The University of Texas MD Anderson Cancer Center, UNITED STATES

## Abstract

Macrophages play a key role in the pathogenesis of liver granuloma and fibrosis in schistosomiasis. However, the underlying mechanisms have not been fully characterized. This study revealed that the macrophages infiltrating the liver tissues in a murine model of Schistosoma japonica infection exhibited M2 functional polarization, and Notch1/Jagged1 signaling was significantly upregulated in the M2 polarized macrophages in vivo and in vitro. Furthermore, the blockade of Notch signaling pathway by a γ–secretase inhibitor could reverse macrophage M2 polarization in vitro and alleviate liver granuloma and fibrosis in the murine model of schistosomiasis. These results implied that the Notch1/Jagged1 signaling-dependent M2 polarization of macrophages might play an important role in liver granuloma and fibrosis in schistosomiasis, and the inhibition of Notch1/Jagged1 signaling might provide a novel therapeutic approach to administrate patients with schistosomiasis.

## Introduction

Schistosomiasis is the second most common parasitic disease in the world, with about 200 million people infected worldwide [1]. In China, in spite of significant progress in controlling Schistosom*a* japonica, there were still more than 300 thousand cases of schistosomiasis reported by the end of 2010. The primary cause of mortality in patients with schistosomiasis is the liver granulomata induced by the eggs of adult worms and subsequent hepatic fibrosis [2–4]. It has been reported that the granulomatous response and fibrosis in the liver continues to worsen, even after effective schistosomicides have been administered [5–7]. Therefore, treatments to reduce or reverse liver granulomata and fibrosis subsequently become the focus of therapeutic strategies.

Studies have shown that in the early stages of schistosomiasis, the host immune responses are of the Th1-type with liver granulomatous inflammation, which shifts to Th2-associated immune suppression in response to the parasite eggs, resulting in the secondary liver fibrosis [8]. To date, however, the mechanism underlying this process has not been fully illuminated. Research has identified that liver macrophage, the main cellular constituent of granuloma, as the key regulator of hepatic fibrosis [9,10]. It has been demonstrated that macrophages can be polarized into the following two major subsets: M1 and M2. The M1 subset consists of classically activated macrophages (CAMs), which are induced by Th1-type cytokines, such as IFN-γ, and up-regulate the expression of nitric oxide synthase-2 (NOS-2). CAMs can activate the adaptive immune response and directly phagocytose pathogens. In contrast, the M2 subset consists of alternatively activated macrophages (AAMs), induced by IL-4 or -13, and up-regulate the expression of arginase-1 (Arg-1) [11, 12]. M2 polarization of macrophages can result in downregulated immune responses [13, 14]. Schistosome eggs can induce AAMs-rich granulomata and Th2-type immunity that prevent acute mortality but subsequently cause hepatic fibrosis [11,15]. Accumulated studies have laid emphasis on the role of Notch signaling pathway in the regulation of macrophage activation and function [16–18]. One study demonstrated that a soluble egg antigen (SEA) of schistosoma could robustly induce the expression of the Notch ligand Jagged1 in mouse and human macrophages, implying that Jagged1 might have a specific function in the process of macrophage M2 polarization [19].

In this study, we used a murine model of schistosomiasis to investigate the role of macrophages in the pathogenesis of liver granulomata and fibrosis. We found that the macrophages infiltrating hepatic tissues exhibited M2 functional polarization, which was dependent on the activation of Notch1/Jagged1 signaling. Interestingly, the inhibition of Notch1/Jagged1 signaling by a γ–secretase inhibitor (GSI) could attenuate liver granulomata and fibrosis via reversing macrophage M2 polarization in the murine model. The results indicated that Notch1/Jagged1 signaling might be a novel target for the treatment of patients with schistosomiasis.

## Materials and Methods

### Cell culture and cytokines assay

RAW264.7 cells were cultured in RPMI 1640 with 10% FBS and incubated at 37°C. ELISA assays were performed with the ELISA Kits (eBioscience, San Diego, CA) to detect IL-10 and IL-12 in the culture supernatants according to the manufacturer’s protocol.

### Animals and treatment

The cercariae were obtained from the Department of Microbiology, Tongji Medical College, Huazhong University of Science and Technology, Wuhan, China. N-[N-[3,5-difluorophenacetyl]-L-alanyl]-S-phenylglycine t-butyl ester (DAPT) was purchased from Sigma-Aldrich Corporation (U.S.) and dissolved in DMSO (Sigma-Aldrich).

6 to 8 week-old female BALB/c mice were obtained from the Experimental Animal Center of Tongji Medical College, Huazhong University of Science and Technology, China. Mice were randomly assigned to 3 groups of 8 mice each as follows: the model group, the DAPT-treated group and control group. In the model and DAPT-treated groups, each mouse was infected percutaneously with 25 Schistosoma japonicum cercariae for 12 weeks. In the DAPT-treated group, each mouse was given DAPT (10 mg/kg) 8 weeks post-infection with cercariae by intraperitoneal injections daily for 4 weeks. Mice in the control group were neither infected with cercariae nor treated with DAPT, but were administered the same volume of solvent.

The mice were fed on a standard diet in the animal house of the Experimental Animal Center of Tongji Medical College, Huazhong University of Science and Technology, China. The animals were kept under conditions of temperature (23–25°C) and relative humidity (50–55%). Mice were monitored closely twice a day. The animal’s activity, nest building, and interaction with cage mates were indicators of general health and well-being. The body condition in mice was assessed by passing a finger over the sacroiliac bones and assigned a score as described previously [20]. All of the mice were sacrificed under anesthesia with isoflurane 12 weeks post-infection with cercariae. Liver tissues were obtained from the mice and preserved for histological analysis in 4% paraformaldehyde.

All of the animal protocols used in this study were performed based on the ethical guidelines of the Animal Care and Use Committee of Huazhong University of Science and Technology, China. This study was approved by the Ethical Committee of Tongji Medical College, Huazhong University of Science and Technology, China.

### Histological examination of the liver

Serial sections of mouse liver tissue were stained with hematoxylin and eosin (HE) to investigate the size of the granulomata. The diameters of the 5 largest granulomas in each section were measured and the mean granuloma diameter were calculated for each group of mice. Only circular granulomatas in the liver tissue section were measured as previously described [21].

The collagen deposition was assessed by Masson's trichrome staining, and the expression of collagen was calculated semi-quantitatively by mean optical density (MOD) using image analysis software (Image Plus Pro 6.0). The MOD was detected for each mouse, and the overall mean was calculated for each group. The total liver hydroxyproline content was detected by the hydrolysates of mouse hepatic specimens in accordance with the instructions for the Hydroxyproline Testing Kit (Jiancheng, China).

### Immunofluorescence staining

Liver specimens fixed in 4% paraformaldehyde were embedded with paraffin. The expression F4/80, CD206 and Arg-1 in mouse liver tissues were detected by immunofluorescence staining. The antibodies used are shown in Table 1. The fluorescent secondary antibodies were conjugated with Alexa Fluor 488 (green) or Alexa Fluor 594 (red) (Invitrogen, CA). The sections were imaged with confocal fluorescence microscope (Nikon, Japan).

**Table 1:** Primary antibodies used for immunofluorescence and Western blot.

Mice antigens	Poly/mono- clonal	Manufacturer	Dilution
F4/80	monoclonal	Abcam, US	1:100
Arg-1	polyclonal	Abcam, US	1:200 (1:1000 for Western blot)
CD206	polyclonal	Abcam, US	1:1000
NOS-2	polyclonal	Abcam, US	1:500 for Western blot
Notch1	monoclonal	Abcam, US	1:2000 for Western blot
Jagged1	polyclonal	Santa Cruz Biotechnology, Inc., CA	1:1000 for Western blot
Hes1	polyclonal	Santa Cruz Biotechnology, Inc., CA	1:100 for Western blot
GAPDH	polyclonal	Santa Cruz Biotechnology, Inc., CA	1:1000 for Western blot

### Preparation of soluble egg antigen (SEA)

SEA was administrated as previously described [21]. Briefly, freeze-dried eggs were mixed with silicon dioxide and PBS. Following centrifugation of the mixture, the supernatant was harvested and ultracentrifuged. The final supernatant was sterilized, and the SEA concentration was detected by the Lowry method.

### Real-time PCR

The total RNA was extracted from cultured RAW264.7 cells or mouse liver tissue using Trizol and converted into cDNA as previously described [17]. Then, the cDNA was subjected to quantitative real-time PCR by the SYBR Green PCR Master Mix (Applied Biosystems, CA), following the instructions. The primers used in the study are listed in Table 2.

**Table 2:** The Primer sequences for real-time PCR.

Notch1	Forward	5‘- TGTGACAGCCAGTGCAACTC—3’
	Reverse	5‘- TGGCACTCTGGAAGCACTGC—3’
Notch2	Forward	5‘- ACATCATCACAGACTTGGTC—3’
	Reverse	5‘- CATTATTGACAGCAGCTGCC—3’
Notch3	Forward	5‘- ATTTCCCATACCCACTTCGG—3’
	Reverse	5‘- CTGTGTTCTCGCTTTCGCCT—3’
Notch4	Forward	5‘- GCAGGGTTTGTGGTAGTG—3’
	Reverse	5‘- GGTGAACATAGAATGGGC—3’
Jagged1	Forward	5'- ATTCGATCTACATAGCCTGTGAG—3'
	Reverse	5'- CTATACGATGTATTCCATCCGGT—3'
Jagged2	Forward	5‘- CTGATTGGCGGCTATTACTG—3’
	Reverse	5‘- GTCGTACTCTAGTTCGCAAT—3’
Hes1	Forward	5'- CCGGTCTACACCAGCAACAGT—3'
	Reverse	5'- CACATGGAGTCCGAAGTGAGC—3'
β-actin	Forward	5’- CACGATGGAGGGGCCGGACTCATC—3’
	Reverse	5’- T AAAGACCTCTATGCCAACACAGT - 3’

### Western blot

Western blot analysis was performed as previously described [22]. Briefly, RAW264.7 cells or mouse liver tissues were lysed and the protein concentrations were detected using the BCA Assay Kit (Beyotime, China). Polyvinylidene difluoride membrane was incubated with the indicated primary antibodies, followed by incubation with the horseradish peroxidase-conjugated secondary antibodies, and final detection was performed by chemiluminescence, using the BeyoECL Plus (Beyotime, China), and exposure to film. The bands were analysed by ImageJ 1.51b (NIH, US). The primary antibodies are shown in Table 1.

### Statistical analysis

The experimental data were described as the mean ± SD from 3 independent experiments. Data were considered significant at *P* < 0.05. The analyses were performed by SPSS 18.0 (IBM, US) and GraphPad Prism 5.0 (GraphPad Software, US).

## Results

### 1. The infiltrated macrophages in liver tissues of murine schistosomiasis show M2 polarization

**Fig 1:**
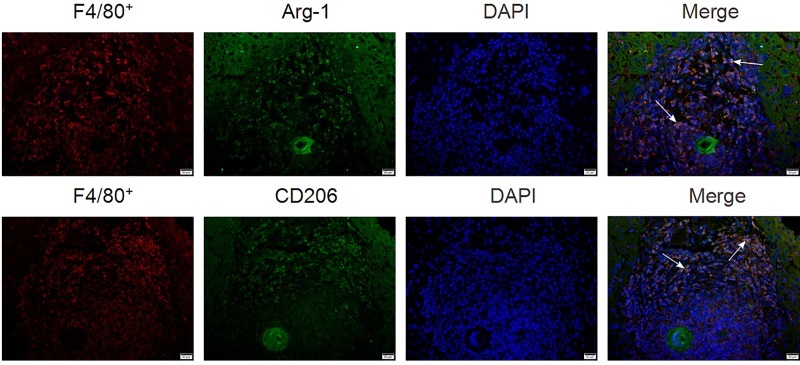
The infiltrated macrophages in liver tissues exhibit M2 polarization in schistosomiasis mice. After eight weeks of cercariae or PBS administration liver sections of mice were stained with antibodies against F4/80^+^, Arg-1 and CD206. Nuclei were stained with DAPI. Images were taken by confocal fluorescent microscopy. Arrows indicate F4/80^+^/Arg-1 or F4/80^+^/CD206 double positive cells, and the bars represent 20 μm.

To investigate the role of macrophages during the pathogenesis of liver fibrosis in a murine model generated by stimulating female BALB/c mice with cercariae of Schistosoma japonicum, the macrophages in the liver granulomata of the schistosomiasis mice were analyzed by immunofluorescence. The results showed that the numbers of F4/80+ cells were markedly increased in granulomata in the liver tissues relative to the liver tissue of normal mice (data not shown). Moreover, the F4/80+ cells also showed co-expression of Arg-1 or CD206, which indicated that the infiltrated F4/80+ macrophages exhibited the M2-polarized phenotype in the murine schistosomiasis model (Fig 1).

### 2. Macrophages treated with SEA display the M2 phenotype in vitro

**Fig 2:**
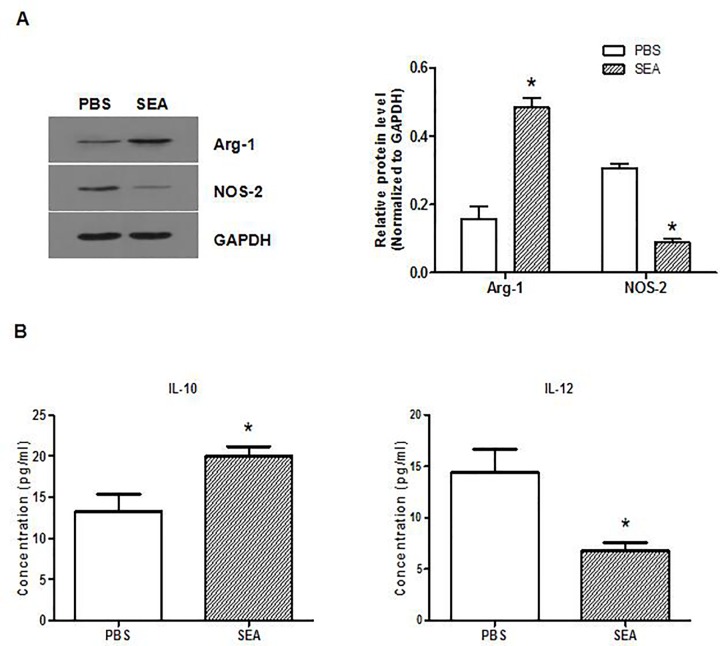
SEA-induced macrophage M2 polarization in vitro. RAW264.7 cells were stimulated with SEA (40 μg/ml) or PBS for two hours. (A) The expression of Arg-1 and NOS-2 in the RAW264.7 cells was assessed by Western blot. (B) The production of IL-10 and IL-12 in the supernatants of RAW264.7 cells was assessed by ELISA. Data represents the results of three independent experiments. In compared with the control group, *: *p* < 0.05.

To further explore whether SEA could induce macrophage M2 polarization, RAW264.7 cells were stimulated with SEA in vitro. The results showed that the expression of enhanced Arg-1 and decreased NOS-2 were observed in SEA-induced macrophages compared to the untreated control cells (Fig 2A). Meanwhile, significantly higher levels of IL-10, but not IL-12, were detected in the supernatants of RAW264.7 cells stimulated with SEA compared to those of the control group (Fig 2B). These data revealed that macrophages induced by SEA showed the M2-polarized phenotype in vitro.

### 3. SEA upregulates Notch1/Jagged1 signaling in M2 macrophages

**Fig 3:**
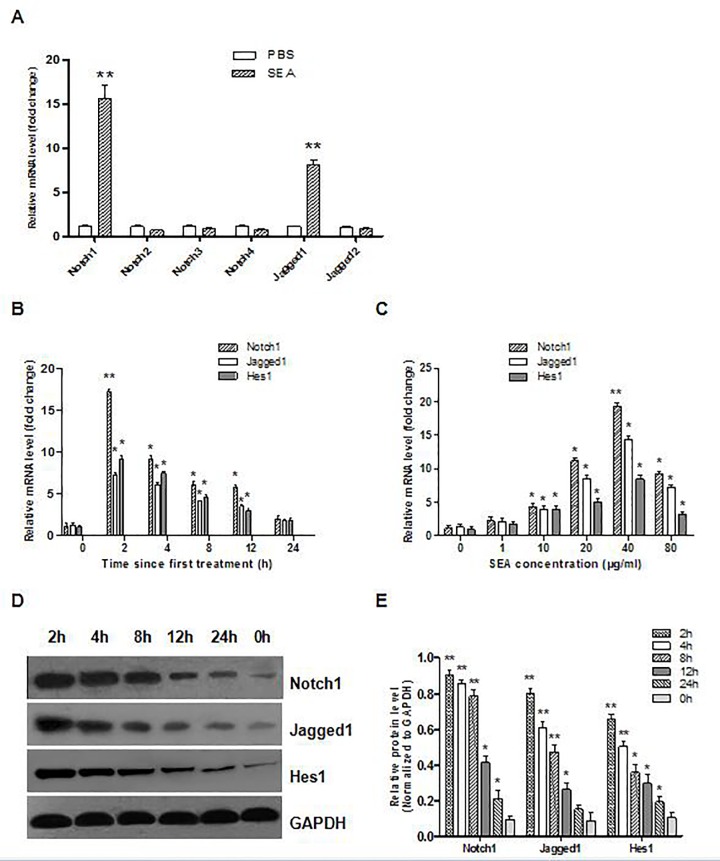
SEA upregulates Notch1/Jagged1 signaling in M2 macrophages in vitro. (A) RAW264.7 cells were treated with SEA (40 μg/ml) or PBS for two hours. The mRNA expression of Notch receptors, Jagged1 and Jagged2 in the RAW264.7 cells were assessed by real-time PCR. (B and C) The mRNA levels of Notch1, Jagged1 and Hes1 in RAW264.7 cells stimulated with SEA (40 μg/ml) for the indicated time or with increasing concentration of SEA for two hours was assayed by real-time PCR. (D and E) The protein expression of Notch1, Jagged1 and Hes1 in RAW264.7 cells treated with SEA (40 μg/ml) for the indicated time was determined by Western blot analysis. Data shown are representative the three independent experiments. In compared with the control group, *: *p* < 0.05, **: *p* < 0.01.

Previous studies had indicated that the Notch signaling pathway facilitated macrophage activation [23–25]. To investigate the expression pattern of Notch receptors in activated macrophages, real-time PCR was used to measure Notch1, -2, -3, -4, Jagged1, and -2 mRNA levels. As shown in Fig 3A, the treatment of RAW264.7 cells with SEA increased the mRNA levels of Notch1 and Jagged1, but not those of Notch2, Notch3, Notch4 or Jagged2 in macrophages. Furthermore, SEA stimulation increased the expression of Notch1, Jagged1 and Hes1 at mRNA and protein levels in a time- and dose-dependent manner in RAW264.7 cells in vitro (Fig 3B, 3C, 3D and 3E).

### 4. DAPT blocks SEA-induced macrophage M2 polarization

**Fig 4:**
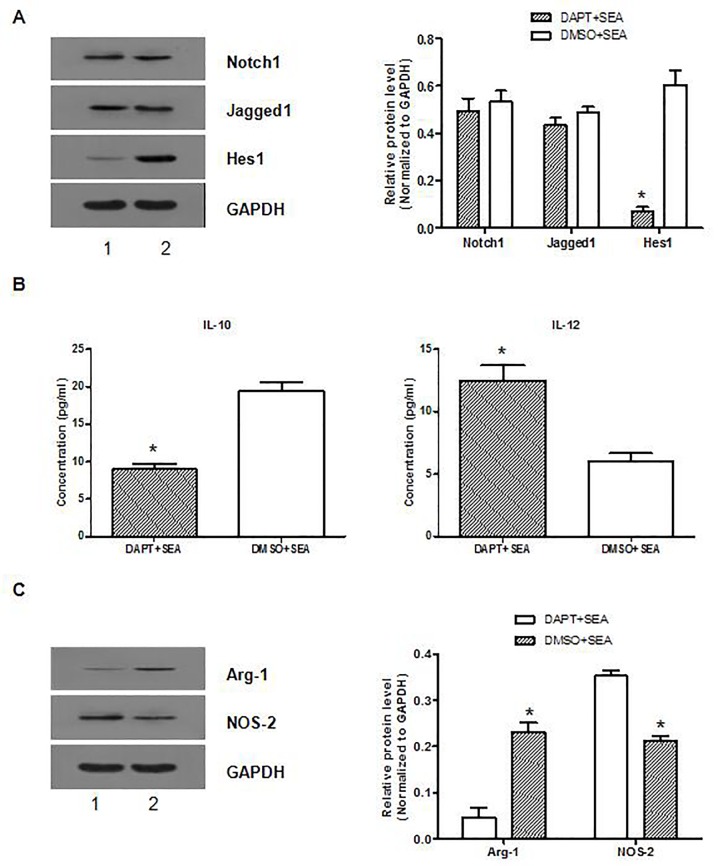
DAPT blocks SEA-induced macrophage M2 polarization by inhibiting Notch signaling. RAW264.7 cells were administrated with DAPT (0.4 μmol/l) or DMSO (0.1%) for twelve hours, and then stimulated with SEA (40 μg/ml) or PBS for two hours. (A) The expression of Notch1, Jagged1 and Hes1 in the RAW264.7 cells was determined by Western blot. (B) The levels of IL-10 and IL-12 in the supernatants were assessed by ELISA. (C) The expression of Arg-1 and NOS-2 in the RAW264.7 cells was assessed by Western blot. All data represent the results of three independent experiments. In compared with the control group, *: *p* < 0.05. 1 indicates DAPT+SEA; 2 indicates DMSO+SEA.

To explore the role of Notch signal in SEA-induced macrophage M2 polarization, we investigated the functional activity of RAW264.7 cells after treatment with DAPT, a γ–secretase inhibitor, and SEA in vitro. Real-time PCR and Western blot analysis showed that DAPT efficiently inhibited Hes1 expression (Fig 4A).

ELISA analysis of inflammatory markers showed significantly decreased production of IL-10, but increased IL-12, in DAPT-treated cells compared to untreated control cells (Fig 4B). Western blot analysis revealed reduced expression of Arg-1 and increased expression of NOS-2 in DAPT-treated RAW264.7 cells relative to the control cells (Fig 4C). These data indicate that the inhibition of Notch1/Jagged1 signaling by DAPT could block SEA-induced macrophage M2 polarization.

### 5. DAPT reverses macrophage M2 polarization and attenuates hepatic granulomata and fibrosis in murine schistosomiasis

**Fig 5:**
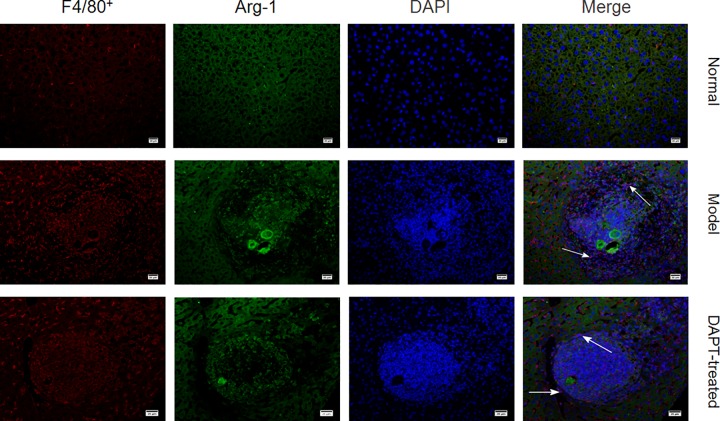
DAPT reverses macrophage M2 polarization in murine schistosomiasis. Mice were infected with cercariae or PBS for twelve weeks. Eight weeks after the initial administration, the mice were treated with DAPT or vehicle control for another four weeks. Twelve weeks after the initial treatment, mice were sacrificed, and the liver sections were stained with antibodies against F4/80^+^ and Arg-1. Nuclei were stained with DAPI. Images were taken by confocal fluorescent microscopy. Arrows indicate F4/80^+^/Arg-1 double positive cells, and the bars represent 20 μm.

**Fig 6:**
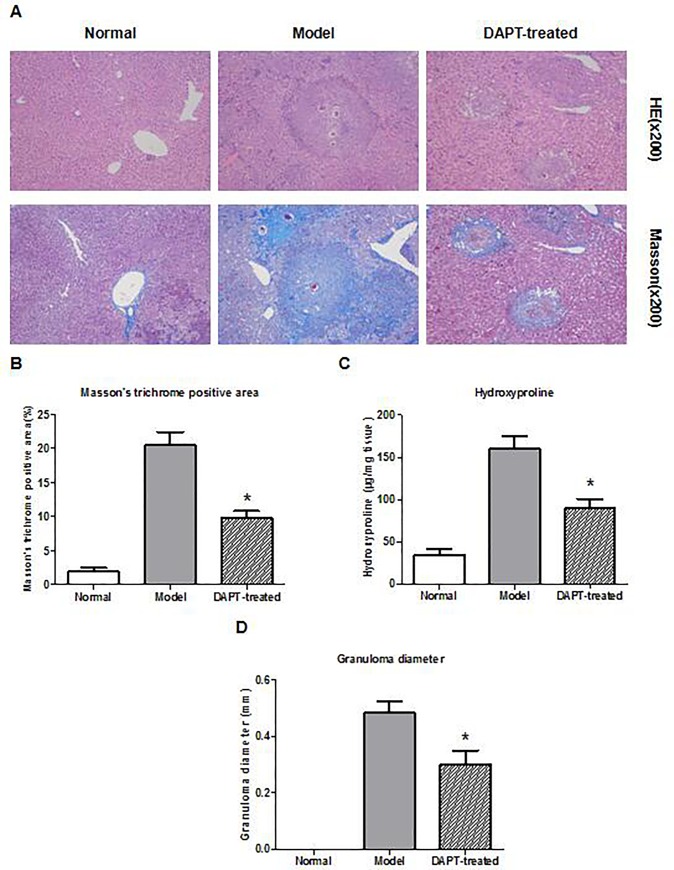
DAPT attenuates hepatic granulomata and fibrosis in murine schistosomiasis. (A) The pathological changes and collagen deposition were assessed by Hematoxylin and eosin and Masson’s trichrome staining. (B) The result of semi-quantitative analysis of the Masson’s trichrome staining. (C) Assay of hydroxyproline content. (D) Mean hepatic granuloma diameter (mm). Data from the three independent experiments represent the results of eight mice in each group. *: *P* < 0.05 versus mice in the model group.

To further investigate whether the inhibition of Notch1/Jagged1 signaling could regulate the status of macrophage polarization and attenuate liver granulomata and fibrosis in murine schistosomiasis, mice were infected with cercariae or PBS for twelve weeks. Eight weeks after the initial administration, the mice were treated with DAPT or vehicle control by intraperitoneal injection daily for another four weeks. The results showed that the expression of Arg-1 decreased obviously in the infiltrated macrophages in liver tissues of the DAPT-treated mice compared to those in control group (Fig 5). Meanwhile, the mean liver granuloma diameter, hydroxyproline content, and collagen deposition was markedly decreased in the DAPT-treated group in compared with those of the untreated schistosomiasis model group (Fig 6).

## Discussion

Schistosoma japonicum causes serious damage to host organs, mainly via the induction of immune responses. Macrophages play critical roles in host defense, immune regulation, and wound healing [1]. Although the role of macrophage activation in the pathogenesis of liver granulomata and fibrosis during Schistosoma japonica infection has been validated, the detailed mechanism of functional macrophage polarization in liver granulomata remains unclear. In this study, we observed the accumulation of AAMs in mouse liver during a Schistosoma japonica infection and identified the effect of DAPT on the AAMs activation via regulation *of* Notch1/Jagged1 signaling in vivo and in vitro.

Schistosome eggs are metabolically active and antigenic and can induce AAMs-rich granulomata that wall themselves off from the surrounding tissues. AAMs function in many pathological processes, especially in repairing the tissue damage caused by parasites [23]. It had been confirmed that Th1 immunity, CAMs, and expression of NOS-2 correlate with acute mortality in schistosomiasis, while Th2 cytokine, AAMs activation, and Arg-1 expression correspond to fibrogenesis. While AAMs are supposed to facilitate wound-healing during acute infection, they result in liver fibrosis during chronic schistosomiasis [11, 15].

In this study, we demonstrated that macrophages stimulated by SEA were activated and M2-polarized and could subsequently induce immune responses against SEA, resulting in liver granulomata and fibrosis in a murine model of a Schistosoma japonica infection. Several evidences supported this opinion. First, the liver granulomata and fibrotic areas of schistosomiasis mice were found to be infiltrated with many activated, M2-polarized macrophages. Second, SEA could induce macrophage M2 polarization in vitro. Furthermore, blocking macrophage M2 polarization by DAPT could decrease the autoimmune response and alleviate liver granulomata and fibrosis in the murine Schistosoma japonica model. All of these results demonstrate that macrophage activation and M2 polarization could facilitate the formation of SEA-induced liver granulomata and fibrosis.

The Notch signal is a highly conserved pathway that regulates cell-fate decisions during embryonic and post-natal development through the interaction with adjacent cells [24]. It has been reported that Notch signaling participates in the cell-fate decision of monocytes and the functional modulation of macrophages [25, 26]. Enhanced Notch1 signaling in macrophages induced by TLRs and various other stimuli could regulate macrophage function, including the production of cytokines, antigen-presenting capacity, and cytotoxic activity [27]. Recently, one study revealed that Notch signal plays a key role in determining M1 versus M2 polarization of macrophage activation [16]. In our present study, significant activation of Notch1/Jagged1 signaling was found in M2-polarized macrophages induced by SEA. Furthermore, we demonstrated that Notch1/Jagged1 signaling contributed to SEA-induced macrophage M2 polarization. Moreover, we found that the inhibition of Notch1/Jagged1 signaling by DAPT could block macrophage M2 polarization and ameliorate liver granulomata and fibrosis in a murine model of Schistosomiasis japonica.

In addition to the previous reports about the effect of Notch1 signaling on the differentiation and activation of T/B cells [24], this study revealed the possibility that Notch1/Jagged1 signaling might participate in the regulation of macrophage activation and polarization in schistosomiasis. It is likely that a GSI treatment could ameliorate established liver granulomata and fibrosis in the murine schistosomiasis model by reversing macrophage M2 polarization. The study revealed that Notch1/Jagged1 signal activation participates in the pathogenesis of liver granulomata and fibrosis in schistosomiasis through the facilitation of SEA-induced macrophage M2 polarization.

Altogether, our present investigation demonstrates that activation of Jagged1/Notch1 signaling regulates SEA-induced macrophage M2 polarization, which exerts a critical role in the pathogenesis of liver granulomata and fibrosis in murine schistosomiasis. These findings imply new therapeutic strategies might be developed that inhibit Notch1/Jagged1 signaling, thereby reversing the M2 polarization of macrophages, which might subsequently attenuate liver granulomata and fibrosis in schistosomiasis.

## References

[1] Colley DG, Bustinduy AL, Secor WE, King CH. Human schistosomiasis. Lancet. 2014; 383(9936): 2253–2264. 10.1016/S0140-6736(13)61949-2 24698483 PMC4672382

[2] Colley DG Secor WE. Immunology of human schistosomiasis. Parasite Immunol. 2014; 36(8): 347–357. 10.1111/pim.12087 25142505 PMC4278558

[3] Chu D, Li C, Wu Q, Shen J. Paeoniflorin prevents hepatic fibrosis of Schistosomiasis japonica by inhibiting TGF-β1 production from macrophages in mice. Frontiers of Medicine in China. 2008; 2(2): 154–165. 10.1007/s11684-008-0029-7

[4] Aly IR, Hendawy MA, Ali E, Hassan E, Nosseir MM. Immunological and parasitological parameters after treatment with dexamethasone in murine Schistosoma mansoni. Mem Inst Oswaldo Cruz. 2010; 105(6): 729–735. S0074-02762010000600001 [pii]. 20944985 10.1590/s0074-02762010000600001

[5] Cioli D, Pica-Mattoccia L, Basso A, Guidi A. Schistosomiasis control: praziquantel forever? Mol Biochem Parasitol. 2014; 195(1):23–29. 10.1016/j.molbiopara.2014.06.002 24955523

[6] Wang W, Wang L, Liang YS. Susceptibility or resistance of praziquantel in human schistosomiasis: a review. Parasitol Res. 2012;111(5):1871–1877. 10.1007/s00436-012-3151-z 23052781

[7] Secor WE, Montgomery SP. Something old, something new: is praziquantel enough for schistosomiasis control? Future Med Chem. 2015;7(6):681–684. 10.4155/fmc.15.9 25996059 PMC4795816

[8] Barsoum Rashad S., Esmat Gamal, El-Baz Tamer. Human Schistosomiasis: Clinical Perspective: Review. J Adv Res. 2013; 4(5): 433–444. 10.1016/j.jare.2013.01.005 25685450 PMC4293888

[9] Tacke F, Zimmermann HW. Macrophage heterogeneity in liver injury and fibrosis. J Hepatol. 2014; 60(5):1090–1096. 10.1016/j.jhep.2013.12.025 24412603

[10] Wynn TA. Barron L. Macrophages: master regulators of inflammation and fibrosis. Semin Liver Dis. 2010; 30(3): 245–257. 10.1055/s-0030-1255354 20665377 PMC2924662

[11] Fairfax K, Nascimento M, Huang SC, Everts B, Pearce EJ. Th2 responses in schistosomiasis. Semin Immunopathol. 2012; 34(6): 863–871. 10.1007/s00281-012-0354-4 23139101

[12] Dewals BG, Marillier RG, Hoving JC, Leeto M, Schwegmann A, Brombacher F. IL-4R alpha-independent expression of mannose receptor and Ym1 by macrophages depends on their IL-10 responsiveness. PLoS Negl Trop Dis. 2010; 4(5): e689 10.1371/journal.pntd.0000689 20502521 PMC2872644

[13] Li K, Xu W, Guo Q, Jiang Z, Wang P, Yue Y, et al Differential macrophage polarization in male and female BALB/c mice infected with coxsackievirus B3 defines susceptibility to viral myocarditis. Circ Res. 2009; 105(4): 353–364. 10.1161/CIRCRESAHA.109.195230 19608981

[14] Liu G, Yang H. Modulation of macrophage activation and programming in immunity. J Cell Physiol. 2013; 228(3): 502–512. 10.1002/jcp.24157 22777800

[15] Caldas IR, Campi-Azevedo AC, Oliveira LF, Silveira AM, Oliveira RC, Gazzinelli G. Human schistosomiasis mansoni: immune responses during acute and chronic phases of the infection. Acta Trop. 2008 Nov-Dec;108(2–3):109–17. 10.1016/j.actatropica.2008.05.027 18577364

[16] Wang YC, He F, Feng F, Liu XW, Dong GY, Qin HY, et al Notch signaling determines the M1 versus M2 polarization of macrophages in antitumor immune responses. Cancer Res. 2010; 70(12): 4840–4849. 10.1158/0008-5472.CAN-10-0269 20501839

[17] Zhang W, Xu W, Xiong S. Blockade of Notch1 signaling alleviates murine lupus via blunting macrophage activation and M2b polarization. J Immunol. 2010; 184(11): 6465–78. 10.4049/jimmunol.0904016. 20427764

[18] Xu H, Zhu J, Smith S, Foldi J, Zhao B, Chung AY, et al Notch-RBP-J signaling regulates the transcription factor IRF8 to promote inflammatory macrophage polarization. Nat Immunol. 2012; 13(7): 642–650. 10.1038/ni.2304 22610140 PMC3513378

[19] Goh F, Irvine KM, Lovelace E, Donnelly S, Jones MK, Brion K, et al Selective induction of the Notch ligand Jagged-1 in macrophages by soluble egg antigen from Schistosoma mansoni involves ERK signalling. Immunology. 2009; 127(3): 326–337. 10.1111/j.1365-2567.2008.02979.x 19019093 PMC2712101

[20] Burkholder T, Foltz C, Karlsson E, Linton CG, Smith JM. Health Evaluation of Experimental Laboratory Mice. Curr Protoc Mouse Biol. 2012; 2: 145–165. 10.1002/9780470942390.mo110217 22822473 PMC3399545

[21] Chu D, Du M, Hu X, Wu Q, Shen J. Paeoniflorin attenuates schistosomiasis japonica-associated liver fibrosis through inhibiting alternative activation of macrophages. Parasitology. 2011; 138 (10): 1259–1271. 10.1017/S0031182011001065 21810309

[22] Chen Y, Zheng S, Qi D, Zheng S, Guo J, Zhang S, et al Inhibition of Notch signaling by a γ-secretase inhibitor attenuates hepatic fibrosis in rats. PLoS One. 2012; 7(10): e46512 10.1371/journal.pone.0046512 23056328 PMC3463607

[23] Allen JE, Wynn TA. Evolution of Th2 immunity: a rapid repair response to tissue destructive pathogens. PLoS Pathog. 2011; 7(5): e1002003 10.1371/journal.ppat.1002003 21589896 PMC3093361

[24] Le Borgne R, Bardin A, Schweisguth F. The roles of receptor and ligand endocytosis in regulating Notch signaling. Development. 2005; 132(8):1751–1762. 10.1242/dev.01789 15790962

[25] Sangphech N, Osborne BA, Palaga T. Notch signaling regulates the phosphorylation of Akt and survival of lipopolysaccharide-activated macrophages via regulator of G protein signaling 19 (RGS19). Immunobiology. 2014; 219(9):653–660. 10.1016/j.imbio.2014.03.020 24775271 PMC4101182

[26] Boonyatecha N, Sangphech N, Wongchana W, Kueanjinda P, Palaga T. Involvement of Notch signaling pathway in regulating IL-12 expression via c-Rel in activated macrophages. Mol Immunol. 2012; 51(3–4):255–262. 10.1016/j.molimm.2012.03.017 22463790 PMC3358353

[27] Palaga T, Buranaruk C, Rengpipat S, Fauq AH, Golde TE, Kaufmann SH, et al Notch signaling is activated by TLR stimulation and regulates macrophage functions. Eur J Immunol 2008; 38(1): 174–183. 10.1002/eji.200636999 18085664

